# From Catheters to Wearables: Advanced Hemodynamic Monitoring and the Emerging Role of PPG‐Based Devices

**DOI:** 10.1155/crp/5632972

**Published:** 2026-11-09

**Authors:** Emil Aliev, Arik Eisenkraft, Dean Nachman

**Affiliations:** ^1^ Hadassah Medical Center and Faculty of Medicine Heart Institute Hebrew University of Jerusalem Jerusalem Israel huji.ac.il; ^2^ Department of Military Medicine Faculty of Medicine Institute for Research in Military Medicine The Hebrew University of Jerusalem Jerusalem Israel huji.ac.il; ^3^ Israel Defense Forces Medical Corps Tel Hashomer Ramat Gan Israel

**Keywords:** ambulatory blood pressure monitoring, cardiac output, remote monitoring, wearable devices

## Abstract

Advanced hemodynamic monitoring, including cardiac output (CO), stroke volume (SV), and systemic vascular resistance (SVR), is important for the management of cardiogenic shock, postoperative patients, and heart failure. Traditional approaches such as pulmonary artery catheterization (PAC) provide comprehensive hemodynamic assessment but are invasive, costly, and associated with procedure‐related risks. This review summarizes established invasive and minimally invasive techniques alongside emerging noninvasive approaches, including pulse contour analysis, bioimpedance, pulse wave transit time, and photoplethysmography (PPG)‐based systems. Particular emphasis is placed on PPG‐based technologies, which combine pulse wave analysis, pulse wave transit time, and advanced signal processing to estimate blood pressure and flow‐related parameters. As a representative case study, the clinical development and evaluation of the Biobeat BB‐613WP wearable are described, including preclinical swine hemorrhagic shock models, intensive care and perioperative validation against invasive reference methods, and ambulatory blood pressure monitoring studies. Available evidence suggests that PPG‐based wearables can achieve clinically acceptable agreement with invasive reference methods in selected populations while offering the potential for continuous, noninvasive, and ambulatory monitoring. However, the available evidence is limited by small sample sizes, single‐center studies, controlled conditions, and limited independent validation. Further multicenter, outcome‐oriented studies are required to establish the clinical role of PPG‐based devices across diverse patient populations and stages of care.

## 1. Advanced Hemodynamic Monitoring

Reliable measurements of vital signs, such as blood pressure and arterial oxygen saturation, serve as a cornerstone for medical care [1]. In clinical practice, hemodynamic monitoring encompasses the measurement of variables describing cardiac performance, vascular function, and blood pressure, which may provide information about changes in cardiovascular status. While conventional vital signs such as heart rate, respiratory rate, body temperature, and oxygen saturation are important components of patient monitoring, they are not, by themselves, considered advanced hemodynamic variables and are therefore outside the primary scope of this review.

The complexity of certain medical scenarios mandates a more in‐depth approach to evaluating cardiovascular function, a practice known as advanced hemodynamic monitoring. This form of monitoring extends beyond conventional measures to encompass intricate parameters like cardiac output (CO), systemic vascular resistance (SVR), and stroke volume (SV), which are essential yet not immediately ascertainable through standard diagnostic techniques [2].

The application of advanced hemodynamic monitoring is particularly critical in acute clinical settings. For instance, patients who suffer from cardiogenic or septic shock benefit from advanced monitoring, enabling timely and targeted therapeutic strategies [3–5]. Furthermore, the utility of advanced hemodynamic monitoring extends into the postoperative care of patients who have undergone significant surgical procedures [6, 7]. In such instances, monitoring aids in the early detection of hemodynamic instability, which, if unaddressed, could precipitate postoperative complications [8].

When looking at the continuum of care, advanced hemodynamic monitoring plays a pivotal role in the management of chronic cardiovascular conditions. In patients with heart failure, for example, the careful balance of fluid volume and monitoring of cardiac function may be integral in optimizing patient outcomes, preventing exacerbations of the disease, and enhancing the quality of care and prognosis [9, 10].

## 2. Accepted Methods for Advanced Hemodynamic Monitoring

Pulmonary artery catheterization (PAC) using the Flow‐directed “Swan‐Ganz” pulmonary artery catheter (SGC) is the gold standard for advanced hemodynamic assessment. The catheter is self‐directed to the pulmonary artery by the force that the flow of blood exerts into an inflatable balloon located at its tip. Most catheters have multilumen structures, connected to several apertures along their length, including at the tip. These apertures allow pressure measurements and draw blood from different cardiac compartments in parallel. The balloon at the tip allows indirect estimation of left atrial pressures by occluding proximal blood flow [11].

PAC also allows for cardiac flow measurements, mainly CO, also used for the calculations of other advanced hemodynamic parameters. Two acceptable methods for CO measurements utilize the SGC. Fick’s method utilizes the quotient of estimated oxygen uptake (VO_2_) and the difference of the arterial and mixed venous oxygen content to calculate CO. The thermodilution technique uses a thermistor at the tip of the SGC, which is inserted from a peripheral vein all the way into the pulmonary artery [12]. 10 mL of cold saline solution is injected into the right atrium from a proximal catheter port. The blood temperature is measured by a thermistor at the catheter tip, which lies within the pulmonary artery, and a computer is used to obtain the TD profile by quantifying the change in blood temperature as it flows over the thermistor surface. The CO computer then calculates the flow (i.e., CO from the right ventricle) using the blood temperature information and the volume and temperature of the injected solution. The injection is normally repeated 3–5 times, and the CO is averaged [12].

While commonly acknowledged as a safe medical device, PAC is not without risks, given its invasive nature. Although the incidence of severe adverse events is relatively low, the possibility of such complications remains a consideration in its use. Early complications of PAC include self‐limiting ventricular arrhythmias and right bundle‐branch block, with the potential for complete heart block in patients with preexisting left bundle‐branch block. Rare incidents include guidewire embolization and catheter knotting, requiring specialist intervention for removal, as well as air embolism presenting with respiratory distress and cardiovascular instability. Pulmonary artery perforation is an infrequent (1:3000 cases) but critical complication, alongside later risks of pulmonary infarction, infection, and thrombosis, underscoring the importance of careful monitoring and preventive measures during PAC management [11, 13, 14].

Pulse contour analysis offers a less invasive technique to estimate CO by utilizing the concept that the aortic pulse pressure correlates directly with SV and is inversely related to aortic compliance [2]. Typically, the pulse contour for estimating CO is derived from a femoral artery catheter, which can be periodically calibrated with thermodilution measurements from a central venous catheter. This method incorporates morphological and demographic factors into the CO calculation [15]. It has shown reasonable concordance with PAC measurements in patients with normal or reduced hemodynamic states, but its reliability diminishes in other clinical conditions [2, 16].

The use of invasive monitoring methods is prone to complications that could endanger patients’ lives [17–19] and that they are technically demanding and usually necessitate intensive care unit (ICU) stays, limiting their use to specific high‐risk patients [20].

Thus, advanced hemodynamic monitoring by noninvasive means might enable thoughtful diagnosis and treatment of unstable patients, and to expand the utilization of advanced hemodynamic monitoring to stable and even ambulatory patients while minimizing morbidity and mortality and maximizing clinical outcome.

Noninvasive cardiac performance assessment methods include transthoracic echocardiography, which provides a direct measurement of cardiac parameters and an intermittent rather than continuous estimation of CO. Nowadays, most Intensive Care specialists have the capability to conduct these evaluations, which have demonstrated an acceptable correlation with PAC measurements in some trials [21]. The Transesophageal Doppler probe, which monitors aortic blood flow to estimate CO, is semi‐invasive and thus only suitable for sedated patients [22]. Other imaging modalities, like magnetic resonance imaging (MRI), can directly quantify cardiac volumes and flows, thereby enabling the calculation of CO with a high degree of correlation to invasive techniques [23].

Table 1 summarizes key clinical studies evaluating invasive, minimally invasive, and noninvasive methods for advanced hemodynamic monitoring, with a focus on efficacy metrics and methodological limitations. As shown, minimally invasive arterial waveform and finger‐cuff systems typically demonstrate PE in the 30%–50% range with reasonable trending ability in hemodynamically stable or moderately unstable patients, but performance deteriorates in profound vasodilation, cirrhosis, or rapidly changing vascular tone. Thoracic bioimpedance and electrical cardiometry show moderate agreement with PAC or PiCCO, yet their accuracy is susceptible to factors such as edema, obesity, arrhythmias, and chest drains, limiting generalizability. PPG‐based systems, including Vitalstream, Corsano, and the device discussed in detail here, generally exhibit small biases and PE around or below 40% in carefully selected cohorts, but their precision remains dependent on peripheral perfusion, motion, and devices’ specific algorithms. The ‘Research limitations’ column underscores that many studies are small, single‐center and sometimes manufacturer‐supported, which may overestimate performance and restrict extrapolation to heterogeneous, high‐acuity populations.

**TABLE 1:** Comparative overview of invasive and noninvasive advanced hemodynamic monitoring modalities.

Technology	Measurement principle	Article	Study design	*N* of subjects	Setting	Intervention/comparison	Efficacy outcomes	Technical limitations	Research limitations
Pulmonary Artery Catheter (PAC)	Thermodilution	Kadosh et al., 2023 [3]	Retrospective observational cohort	13,618 admissions (3827 cardiogenic shock)	Cardiac ICU	PAC vs no PAC	PAC use associated with reduced mortality (adjusted OR 0.79)	Invasive	Observational design; confounding bias; registry data limitations
		Binanay et al., 2005 [4]	Randomized controlled trial	433 patients	26 heart failure centers in the US and Canada	PAC vs clinical assessment alone	No significant effect on days alive out of hospital or mortality; PAC group had more in‐hospital adverse events (21.9% vs 11.5%, *p* = 0.04)	Trial stopped early; unblinded; no precise strategy defined for PAC data response	Excluded patients where PAC seemed urgent; results apply to experienced centers; no info on cardiogenic shock/LVAD/transplant triage
		Bertaina et al., 2022 [24]	Meta‐analysis	6 observational studies, 1,166,762 patients	Cardiogenic shock (ICUs)	PAC vs no PAC	Meta‐analytic PAC effect: pooled OR 0.71 (lower mortality), MCS use significantly higher in PAC group (OR 1.60)	Invasive	Heterogeneity among studies; observational data; publication bias
		Ju et al., 2025 [25]	Retrospective cohort (national database)	61,405 patients	Cardiac surgery	PAC vs no PAC	PAC associated with improved 1‐year survival (OR 0.81), more pronounced after off‐pump CABG (OR 0.54) and in less experienced centers (OR 0.70)	Invasive	Retrospective; unmeasured confounders; selection bias
		Watanabe et al., 2024 [26]	Retrospective cohort	83,170 ACS patients (4668 PAC)	Cardiac ICU/ACS (hospitals)	PAC vs no PAC	No mortality benefit; PAC ↑ bleeding and LOS	Invasive	Selection bias; residual confounding; older patient cohort

Arterial line sensor	Direct Arterial pressure waveform analysis	Murata et al., 2022 [27]	Prospective observational	20 patients	OR (liver transplant)	FloTrac (4th gen) vs PAC thermodilution	Correlation *r* = 0.48; bias +1.62 L/min; percentage error (PE) 54.36%; concordance rate 49.39%	Minimally invasive, Sensitive to SVR changes	Small sample; single center; limited CO range; nonstandardized anesthesia management
		Asamoto et al., 2017 [28]	Prospective observational	20 patients	OR (Off‐pump coronary artery bypass graft (OPCAB) and living‐donor liver transplantation (LDLT))	LiDCOrapid vs FloTrac/Vigileo vs PAC	LiDCO bias −0.38 L/min (PE 53.5%, concordance 92.4%); FloTrac bias −0.44 L/min (PE 74.4%, concordance 84.0%)	Minimally invasive, High percentage error; low agreement; tended to underestimate at high Cis	Small, mixed cohort; no calibration; limited stats; consignment study (though authors state no influence)
		Mukkamala et al., 2021 [29]	Prospective observational	58 patients	OR (OPCAB)	Argos vs FloTrac vs PAC	Argos RMSE 1.16 L/min (concordance 87%); FloTrac RMSE 1.54 L/min (concordance 72%); Argos more accurate for trending and absolute CO	Minimally invasive; Device‐specific algorithm; percentage error ∼50% for both	Single‐center; novel device; manufacturer influence
		Senn et al., 2009 [30]	Prospective comparative study	50 patients (25 software A, 25 software B)	ICU (postcardiac surgery)	FloTrac/Vigileo (initial FCOA vs modified FCOB) vs PiCCO plus vs intermittent thermodilution (ICO)	FCOA: bias −0.2 L/min, PE 25%, LOA ± 1.4 L/min	Minimally invasive, Initial software had deficits	small sample; single‐center
							Modified algorithm (FCOB): −0.2 L/min, PE 25.5%, LOA ± 1.3 L/min		
		Biancofiore et al., 2009 [31]	Prospective observational	29 patients (290 pairs)	Liver transplant surgery and ICU	Uncalibrated FloTrac/Vigileo vs PAC	Bias 1.3 L/min/m^2^; PE 54%; concordance 68%,LOA −1.5 to 4.1 L/min/m^2^, *r* 0.69	Minimally invasive; low peripheral resistance related to increasing bias; failed to reliably trend	Not suitable for cirrhotic patients with hyperdynamic circulation

Abbreviations: CABG, coronary artery bypass graft; CO, cardiac output; CPB, cardiopulmonary bypass; LOA, limits of agreement; LOS, length of stay; MCS, mechanical circulatory support; OR, odds ratio; PAC, pulmonary artery catheter; PE, percentage error.

## 3. Novel Noninvasive Monitoring of Advanced Hemodynamics

As noted earlier, though patients’ assessments can be performed using echocardiography and cardiac MRI, invasive arterial and pulmonary capillary wedge pressure monitoring are considered the gold standard of hemodynamic monitoring and may be required in seriously ill ICU patients, as well as in patients with decompensated HF, providing important indications of physiological changes and early recognition of patient deterioration. However, invasive techniques necessitate ICU settings and are associated with inherent risks that hinder their wider usage [17, 32]. Furthermore, noninvasive imaging‐based assessment gives only a single point measurement and may be labor intensive. These downsides of currently available advanced hemodynamic monitoring alongside the shift towards ambulatory and preventive medical care led to the need for developing novel monitoring techniques. The following paragraphs will shed light on some of the more promising technologies.

When it comes to examining the reliability of a device for measuring advanced hemodynamics, the ±30% limits of agreement with thermodilution for cardiac output proposed by Critchley and Critchley are widely accepted [33] and commonly applied [1, 30, 31, 34–37]. Though relatively accepted, Peyton and Chong provided a meta‐analysis of papers [38] that questioned this criterion, showing that thermodilution itself offers no better precision than minimally invasive methods under unstable hemodynamic conditions. Their findings revealed similar limits of agreement among various techniques, implying a fundamental limitation in achievable precision regardless of the underlying measurement principle. They suggested that a 45% limit of agreement with thermodilution more accurately reflects the realistic precision attainable in clinical practice, yet these thresholds remain debated and may depend on the clinical context and methodology used.

### 3.1. Noninvasive Pulse Wave Analysis (PWA)

As previously mentioned, PWA for advanced hemodynamic monitoring can be conducted using several invasive approaches. Recently, the development of methods to noninvasively record arterial pulse wave at high precision has allowed for the estimation of SV, subsequently facilitating the calculation of CO [39, 40].

The Finger Cuff method (Figure 1), often referred to as the Vascular Unloading or Volume Clamp technique, provides an estimate of the arterial pulse wave by utilizing an automated finger cuff. This device measures the dynamic pressure changes required to keep the volume of blood within the arteries of the finger constant. It employs photoplethysmography (PPG), a technique that detects blood volume changes based on light absorption, to continuously adjust the cuff’s pressure. By doing so, it maintains a constant arterial volume, despite the natural fluctuations that occur with each cardiac cycle [39, 41]. Devices currently available on the market have undergone evaluation in multiple clinical studies, yielding inconclusive results regarding their efficacy in assessing cardiac output, particularly when benchmarked against the PAC based technique [42, 43].

**FIGURE 1:**
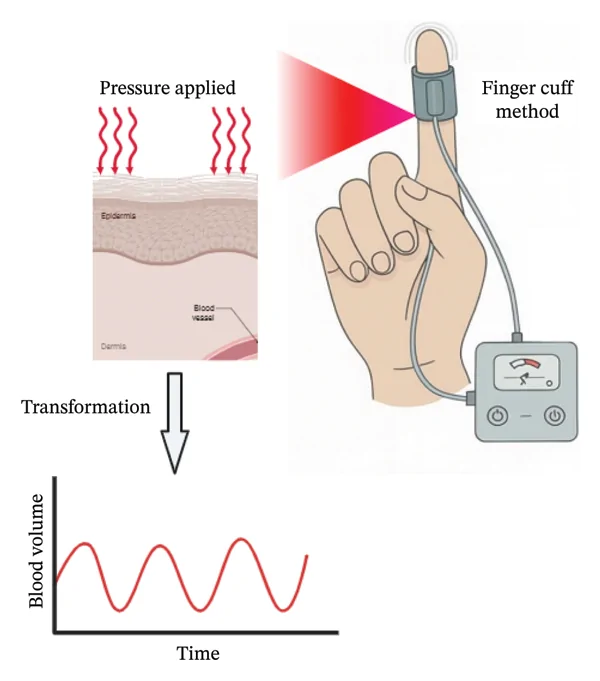
Finger cuff method illustration‐estimates arterial blood pressure and pulse waveform by applying controlled external pressure around the finger, while an optical sensor continuously records volume changes over time and transforms them into a calibrated hemodynamic signal.

Radial artery applanation tonometry (Figure 2) is a technique that involves flattening (applanating) a superficial artery (usually the radial artery) positioned over a bony prominence using an ultrasensitive pressure sensor. This method neutralizes the transmural pressure of the artery, allowing for the capture of the maximal pulse pressure waveform, and CO is then calculated [44]. Wrist watches and hand‐held devices are used to apply this method. Studies comparing the measurements of applanation tonometry‐based devices to CO assessment by echocardiography and PAC demonstrated reasonable agreement [45], mainly when tested on perioperative patients [46]. However, these devices possess an intrinsic limitation: their readings are heavily dependent on the correct and precise placement and are highly susceptible to motion artifacts.

**FIGURE 2:**
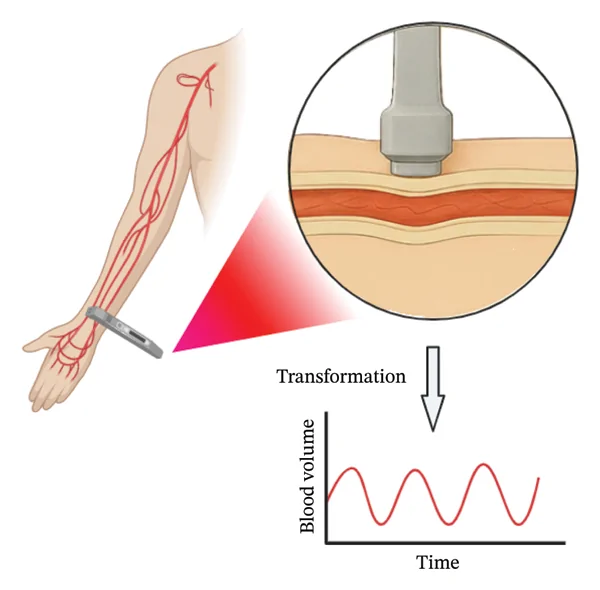
Radial artery applanation tonometry illustration‐pressure sensor flattens the radial artery to capture the arterial pulse waveform and derive blood volume changes over time.

### 3.2. Electrical Bioimpedance

Thoracic electrical bioimpedance posits that the cardiac cycle’s effect on intrathoracic blood volume alters the thorax’s electrical conductivity and impedance, primarily reflecting aortic volume fluctuations (Figure 3). Electrodes placed on the thorax detect these impedance variations by measuring the disparity between an applied voltage and the voltage detected, using a low‐amplitude, high‐frequency current. These impedance shifts are proportional to SV, which can be estimated through the ventricular ejection time combined with the rate of change in aortic volume [47, 48]. This technique carries an inherent risk of bias stemming from its sensitivity to several factors. Patient movement, arrhythmias, and alterations in body composition, such as obesity or the presence of a thoracic aortic aneurysm, can all significantly affect the accuracy of the measurements. These variables can introduce discrepancies in data interpretation, necessitating careful consideration and calibration for individual patient characteristics to ensure reliable results [39]. Clinical validation trials demonstrated a moderate agreement of the bioimpedance method to other methods with a percentage error of 37%–42% [49].

**FIGURE 3:**
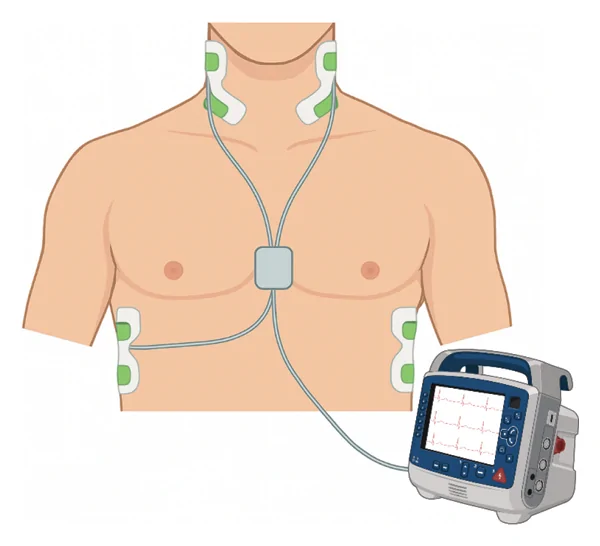
Thoracic electrical bioimpedance illustration‐electrodes placed on the neck and thorax connected to a monitor that measures changes in transthoracic electrical impedance to estimate stroke volume and cardiac output.

### 3.3. Pulse Wave Transit Time (PWTT)

PWTT (Figure 4) is a physiological parameter that quantifies the temporal interval for the arterial pulse wave to traverse from the left ventricular outflow tract to the peripheral arterial sites. This metric is determined by capturing the interval from the peak of the R wave on an electrocardiogram (ECG) to the initial deflection of the pulse wave observed at a peripheral location, such as the radial or femoral artery, by plethysmography or tonometry. The utility of PWTT lies in its reflection of arterial compliance, where reduced transit times are suggestive of increased arterial stiffness. SV and CO can be correlated by combining demographic and anthropomorphic data [39].

**FIGURE 4:**
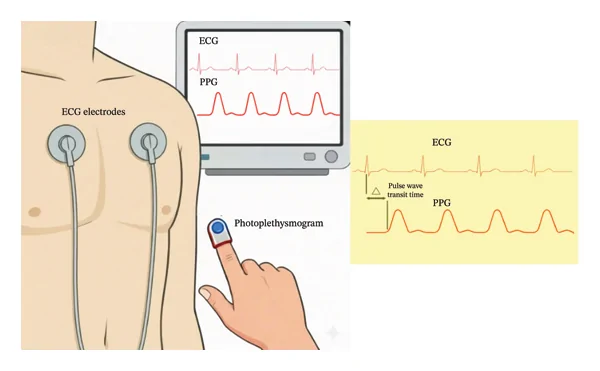
Pulse wave transit time illustration‐simultaneous ECG and PPG recording, with the time delay between the R‐wave and the upstroke of the PPG waveform representing pulse wave transit time.

The application of PWTT for the estimation of CO, however, has limitations. Its accuracy is compromised in patients with cardiac arrhythmias, where the irregularity of heartbeats leads to inconsistent PWTT measurements. Similarly, patients with significant changes in peripheral vascular resistance may also exhibit altered PWTT. These alterations can distort the relationship between PWTT and SV, leading to unreliable CO estimations. Within clinical trial settings, PWTT monitors has not shown satisfactory concordance with thermodilution techniques for measuring CO in patients undergoing cardiac surgery.

Recent advances in novel technologies now provide noninvasive monitoring, potentially offering alternatives [50], yet they show wide percentage error when compared with invasive methods, and their exact role in patient monitoring is still to be fully defined [38, 46, 51–53].

## 4. PPG‐Based Advanced Hemodynamic Monitoring

PPG is a noninvasive optical monitoring technique that measures the blood volume changes in the microvascular bed of tissue (Figure 5). It utilizes an infrared light source and a photodetector placed on the skin’s surface to detect variations in light absorption, which correspond to pulsatile changes in blood volume with each cardiac cycle. These fluctuations in light absorption are used to construct a photoplethysmogram, a waveform that reflects the arterial pulse and is used to provide information about the cardiovascular system. The technique is commonly applied using probes placed on the fingers, toes, or earlobes and is integral to devices such as pulse oximeters, which estimate oxygen saturation levels in addition to heart rate [54].

**FIGURE 5:**
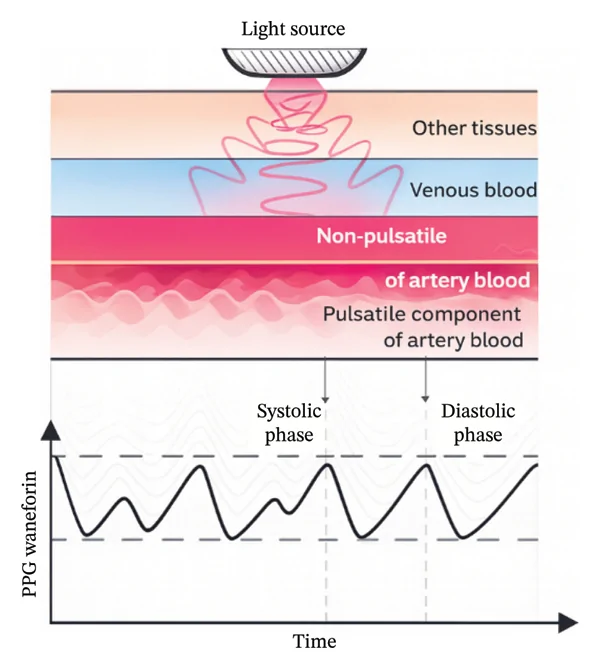
PPG waveform through tissue layers across the cardiac cycle.

PPG technology not only provides valuable data on heart rate and blood oxygen levels but also has potential applications in assessing other hemodynamic parameters such as CO, blood pressure, and vascular resistance. Advanced algorithms and signal processing techniques that incorporate PWTT and PWA are applied to the PPG waveform to extract features that correlate with these physiological parameters [55].

While PPG is advantageous for its simplicity, ease of use, and noninvasiveness, it is sensitive to motion artifacts and variations in probe placement, which can affect the accuracy of the measurements. Additionally, body composition, such as peripheral edema and fat, may alter the readings [54]. Of note, previous studies demonstrated that non‐White skin tone and obesity may influence the reliability of the readings [56]. Ongoing research is focused on enhancing the robustness and reliability of PPG‐based monitoring systems, expanding their use in both clinical and home monitoring settings.

## 5. The Biobeat BB‐613WP Monitor

At this stage of the review, we will focus on a case study of PPG technology that was investigated by the authors of the paper. The PPG‐based advanced hemodynamic monitor, which the authors of the paper were involved in developing, is the BB‐613WP (Biobeat Technologies Ltd., Petach‐Tikva, Israel) and serves as a case study illustrating the advantages and disadvantages of this technology.

This device has been evaluated in preclinical and clinical studies for blood pressure and CO‐related parameters [57]. This monitor is endowed with a comprehensive sensor suite, facilitating the unobtrusive and simultaneous surveillance of an extensive array of physiological metrics. Integral to this device are thermal sensors, an accelerometer, an ECG electrode sensor, and a PPG monitor, each contributing to a full assessment of patient health.

The device is characterized by its fully wireless operation and capability for seamless data transmission. This monitor, devoid of any tethering cables, leverages a local network router or standard mobile devices as conduits for data relay to the clinical team. The data thus transmitted is subjected to rigorous analysis via a monitoring software suite, which is imbued with artificial intelligence (AI) algorithms (Figure 6). These algorithms are adept at discerning patterns within the physiological data, detecting subtle variations, and promptly identifying instances of acute exacerbations, thereby facilitating timely clinical interventions.

**FIGURE 6:**
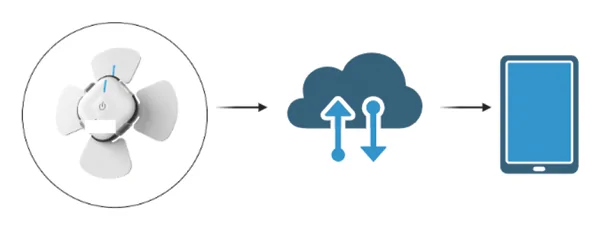
PPG device transmitting physiological data wirelessly to mobile devices and a cloud‐based analytics platform for further analysis.

From a design perspective, the monitor is compact, manifesting as a square with a mere half‐centimeter thickness and a dimension of 3 cm along each side. This design is included in two distinct configurations tailored to patient comfort and monitoring needs: a chest‐mounted adhesive patch variant and a wrist‐worn model. The chest‐mounted version incorporates a battery capable of sustaining continuous operation for up to 5 days; the wrist‐worn configuration integrates a rechargeable battery solution.

Beyond the ambit of conventional monitoring indices such as body temperature, respiratory rate, and cardiac rhythm, this device ventures into the analysis of more intricate and prognostically significant parameters. These include cuffless arterial blood pressure, heart rate variability, and daily activity levels. Moreover, the device is equipped with sophisticated analytical algorithms capable of deciphering complex physiological data, thereby extending its utility to the quantification of SV, CO, and SVR.

The capability of the monitor to accurately track advanced hemodynamic indices is attributed to the synergistic integration of a sophisticated PPG system and advanced computational algorithms. The PPG sensor employed by this device is distinguished by its utilization of reflective technology, wherein the light‐emitting diodes (LEDs) and photodetectors are situated on the same plane. This configuration enables the effective monitoring of broader and bulkier anatomical areas, transcending the limitations of traditional PPG sensors that are typically confined to monitoring smaller, cylindrical appendages such as fingers and ear lobes. Furthermore, the device boasts a temporal resolution in its measurements, achieving a high rate of readings per second. This high‐frequency data acquisition enables the capture of nuanced details within the pulse wave, facilitating a robust analysis of its various characteristics with a high degree of confidence. The sensor leverages advanced PWA technique and PWTT measurements to derive advanced hemodynamic parameters from the pulse wave. Through this methodology, it is capable of calculating blood pressure and SV, which serve as foundational elements for the subsequent computation of more complex hemodynamic indices.

### 5.1. Research and Development to Date

The development of the monitoring device has been underpinned by a comprehensive research and development program, encompassing investigations utilizing large animal models as well as multiple clinical trials. Subsequent to the rigorous evaluation through these studies, the device has attained regulatory approval from the Food and Drug Administration (FDA) and the Conformité Européenne (CE) for specific hemodynamic parameters assessed during the research phase.

In a controlled hemorrhagic shock study on a swine model, the accuracy and validity of the monitoring device were assessed against traditional invasive methods using arterial line (AL) and PAC. Over a median monitoring duration of 447 min, and across 1312 paired observations collected from 11 animals, the PPG device demonstrated concordance with invasive measures, particularly in tracking dynamic changes as bleeding and subsequent hemodynamic shock ensued. Both CO and blood pressure showed strong correlation coefficients (*r* > 0.88) and concordance rates of up to 86% [58].

Clinical studies first focused on the validation of the monitor’s capabilities in measuring blood pressure against conventional devices. In a study involving 1057 participants at public blood pressure screening stations, the PPG device was compared to a standard sphygmomanometer, showing a mean difference in systolic and diastolic measurements of −0.1 ± 3.6 mmHg and 0.0 ± 3.5 mmHg, respectively, placing it within the acceptable range. The devices exhibited a 96.31% agreement in hypertension detection and high interclass correlation coefficients (0.99 for systolic and 0.97 for diastolic measurements), indicating substantial reliability and agreement [57]. As previously described, non‐White skin tone and weight constitute a factor for unreliable measurements in many PPG devices, and in light of this a stratification of these results was performed according to weight, skin color and gender. These variables did not change the high level of validity and accuracy of the monitoring device [56].

In another trial, the continuous measurements of the PPG monitor were compared to invasive AL in ten postcardiac surgery patients over an average duration of 432 ± 290 min. Strong and significant correlations (*p* < 0.001) were observed with correlation coefficients ranging from 0.93 to 0.99 for both the wrist‐worn and the chest‐patch configurations, across approximately 4000 data points. Bland–Altman analysis revealed a marginal bias of less than 1 mmHg for blood pressure and less than 1 beat/min for pulse rate. The LOA of −10, 10 in SBP and −5, 5 in MAP demonstrated relatively high agreement with AL measurements [59]. In another study involving 28 participants, the PPG device was compared to 24 h ambulatory blood pressure monitoring (ABPM). Bland–Altman analysis revealed a 24 h bias of −1.1 mmHg for both systolic and diastolic blood pressure, while receiver operating characteristic (ROC) curve analysis demonstrated excellent diagnostic performance for hypertension classification, with area under the curve (AUC) values reaching up to 1 for systolic measurements. Regarding LOA, the wrist monitor has shown a −4.2, 1.9 mmHg for SBP. The wrist‐worn device was found to cause significantly less inconvenience compared to the cuff‐based device, indicating its potential for more comfortable and effective long‐term blood pressure monitoring [60].

Concurrent with clinical trials evaluating blood pressure measurements and following the swine model study that exhibited high concordance in advanced hemodynamic assessments, preliminary clinical evaluations of the PPG monitor performance in measuring advanced hemodynamic parameters against established invasive devices were undertaken. In a preliminary study involving patients admitted to the general ICU, CO was concurrently monitored using a standard invasive monitoring device and the PPG‐based chest patch. In seven patients (69 paired measurements in total), mean CO values were closely aligned between the thermodilution (7.3 ± 2.0 L/min) and PPG methods (7.0 ± 1.5 L/min), with Bland–Altman analysis revealing a modest bias of 0.3 L/min and a high correlation coefficient (*r* = 0.906) between the two techniques [61].

A recent study of 90 patients evaluated PPG compared to invasive thermodilution PAC and the Fick method. The analysis revealed that the limits of agreement for CO measurement between the PPG device and thermodilution were consistently below the 30% threshold, with values between 14.8% and −19.4% [62]. Similarly, when compared with the Fick method, the limits of agreement remained under 30%, indicating a substantial correlation with the invasive measurements. Notably, the agreement limits between the two invasive techniques themselves surpassed the 30% mark (41.8%, −34.8%). These findings are in concordance with previous comparative studies of these methods that found estimated error as high as 40%–65%, setting the clinically acceptable LOA [34, 63–65]. Further analysis of the additional hemodynamic index (SV, CI, and SVR) measurements yielded a coherent pattern. The agreement limits for comparisons between the PPG device and thermodilution remained beneath the ±30% threshold, except for a marginal exceedance in the lower limit for SV measurements, which registered at −30.5%. In contrast, all comparisons between the Fick method and TD exceeded the ±30% threshold and were notably higher than those observed when comparing the PPG device with each of the invasive methods.

As detailed, this technology has been evaluated in several studies in the past, showing similar results to the current evaluation with limits of agreement as low as 25% and a small bias (0.42 L/min) compared to PAC in a swine model of controlled hemorrhagic shock [58]. The PPG‐device also demonstrated high correlations of CO measurements compared with pulse contour CO (PiCCO) in a small number of septic patients in the general ICU [61]. The results of the current study add more to the confidence of using the PPG‐based device to monitor advanced hemodynamic parameters, including SV, CO, CI, and SVR, especially as it is noninvasive and provides continuous measurements, helping to improve monitoring of patients with complex cardiovascular conditions alongside other clinical scenarios.

Considering previous reports on the lower validity of PPG‐based devices in measuring arterial oxygen saturation in darker skin tones and inaccurate measurements with overweight and obesity, a comparison of the results was made according to these subgroups. It was found that in all the subgroups according to skin tone and BMI, the results maintain a high validity that meets the criteria that were set. These findings contribute to the generalizability of the research results to diverse population groups.

### 5.2. Potential Bias and Independence of Evidence

Potential sources of bias and the independence of the evidence supporting PPG‐based hemodynamic monitoring should be considered when interpreting the available literature. Several studies evaluating PPG‐based devices, including the BB‐613WP system, were conducted in relatively small cohorts, at single centers, and under controlled clinical conditions. In addition, some studies were manufacturer‐supported or involved investigators with direct involvement in device development, which may introduce potential conflicts of interest or other sources of bias and may limit the generalizability of the findings.

For the BB‐613WP specifically, the authors of this review were directly involved in the development and evaluation of the device, and two authors are paid consultants to Biobeat Technologies Ltd.; however, no payment was received for the writing of this review. The clinical studies of the PPG‐based system in which the authors were involved demonstrated favorable agreement with invasive reference methods, but this direct involvement should be considered when interpreting these findings.

## 6. Conclusions

Advanced hemodynamic monitoring has evolved from fully invasive PAC‐based thermodilution to a spectrum of minimally invasive and noninvasive techniques that collectively broaden the ability to characterize cardiovascular status across care settings. While PAC remains the reference standard for measuring CO and derived indices, offering continuous data and rich hemodynamic profiling, it comes at the cost of central venous and arterial cannulation, procedure‐related complications, the need for ICU‐level staffing, and limited suitability for lower‐risk or ambulatory patients. Minimally invasive systems such as pulse contour reduce some procedural risk and provide beat‐to‐beat information, yet still require arterial or central access, frequent calibration, and may lose accuracy in states of vasoplegia, arrhythmia, or rapidly changing vascular tone.

Minimally invasive arterial waveform, finger‐cuff, tonometry, bioimpedance, and PWTT‐based monitors reduce procedural risk and enable beat‐to‐beat or frequent trending, yet their accuracy is influenced by vascular tone, arrhythmias, body habitus, and local perfusion, and many devices show percentage errors around or above the 30%–45% range when compared with thermodilution.

Against this background, PPG‐based wearables represent a further step towards noninvasive, continuous, and potentially ambulatory advanced hemodynamic monitoring. Studies with various PPG‐based devices, including finger‐cuff systems and chest‐ or wrist‐worn sensors, suggest that in selected perioperative, ICU, and ambulatory cohorts, they can achieve small biases and acceptable limits of agreement for BP and CO compared with PAC, PiCCO, and other invasive references, while offering the practical advantages of cable‐free operation and remote data transmission.

The BB‐613WP system illustrates this potential in preclinical swine hemorrhagic shock experiments; ICU validation against thermodilution and ambulatory BP monitoring studies have demonstrated strong correlations and limits of agreement that fall within or close to accepted thresholds for clinical use, including in subgroups defined by skin tone and BMI. However, these data are derived from relatively small, often single‐center studies, many involving relatively controlled conditions and limited patient heterogeneity, and PPG performance may be challenged in severe shock, extreme vasoconstriction, arrhythmias, or extreme body habitus.

The clinical studies of the PPG‐based system, in which the authors were directly involved in both design and execution, showed good correlation between PPG‐derived CO and SVR and invasive reference methods, but they also underscore important constraints and the need for further evaluation in heterogeneous, high‐acuity populations and real‐world ambulatory settings. Overall, the PPG‐based monitoring device currently provides CO and SVR measurements that align well with gold‐standard invasive methods yet should be regarded as a complementary tool rather than a full replacement. With additional studies, this technology may support management of complex clinical scenarios in which invasive monitoring is less desirable or not feasible and may help extend advanced hemodynamic monitoring to ambulatory patients, thereby improving the precision and personalization of care.

**Nomenclature**
CO: Cardiac output
SV: Stroke volume
PAC: Pulmonary artery catheterization
SGC: Swan‐Ganz catheter
VO_2_
: Oxygen uptake
TD: Thermodilution
PWA: Pulse wave analysis
PWTT: Pulse wave transit time
ECG: Electrocardiogram
PPG: Photoplethysmography
AI: Artificial intelligence
LEDs: Light‐emitting diodes
AL: Arterial line
ABPM: Ambulatory blood pressure monitoring
ROC: Receiver operating characteristic
AUC: Area under the curve
ICU: Intensive care unit
HF: Heart failure
MRI: Magnetic resonance imaging
SVR: Systemic vascular resistance
CI: Cardiac index
LOA: Limits of agreement
PiCCO: Pulse contour cardiac output

## Author Contributions

Conceptualization D.N. and A.E.; writing–original draft preparation, E.A. and D.N.; writing–review and editing, D.N. and A.E.; visualization, and E.A., supervision, D.N.

## Funding

This research received no external funding.

## Disclosure

All authors have read and agreed to the published version of the manuscript.

## Ethics Statement

The authors have nothing to report.

## Consent

The authors have nothing to report.

## Conflicts of Interest

Dr. Dean Nachman and Prof. Arik Eisenkraft are paid consultants of Biobeat Technologies Ltd. However, no payment was received for the writing of the article, which was conducted based on their expertise in the topic. The other author declares no conflicts of interest.

## Data Availability

The data that support the findings of this study are available from the corresponding author upon reasonable request.

## References

[1] Pour-Ghaz I. , Manolukas T. , Foray N. et al., Accuracy of Non-Invasive and Minimally Invasive Hemodynamic Monitoring: Where do We Stand?, Annals of Translational Medicine. (2019) 7, no. 17, 10.21037/atm.2019.07.06. PMC6787372 31660320

[2] Kaufmann T. , van der Horst I. C. C. , and Scheeren T. W. L. , This is Your Toolkit in Hemodynamic Monitoring, Current Opinion in Critical Care. (2020) 26, no. 3, 303–312, 10.1097/mcc.0000000000000727. 32332285

[3] Kadosh B. S. , Berg D. D. , Bohula E. A. et al., Pulmonary Artery Catheter Use and Mortality in the Cardiac Intensive Care Unit, JACC Heart Failure. (2023) 11, no. 8 Pt 1, 903–914, 10.1016/j.jchf.2023.04.007. PMC10527413 37318422

[4] Binanay C. , Califf R. M. , Hasselblad V. et al., Evaluation Study of Congestive Heart Failure and Pulmonary Artery Catheterization Effectiveness: the ESCAPE Trial, JAMA. (2005) 294, no. 13, 1625–1633. 10.1001/jama.294.13.1625 16204662

[5] Lee E. P. , Wu H. P. , Chan O. W. , Lin J. J. , and Hsia S. H. , Hemodynamic Monitoring and Management of Pediatric Septic Shock, Biomedical Journal. (2022) 45, no. 1, 63–73, 10.1016/j.bj.2021.10.004. PMC9133259 34653683

[6] Funk D. J. , Moretti E. W. , and Gan T. J. , Minimally Invasive Cardiac Output Monitoring in the Perioperative Setting, Anesthesia & Analgesia. (2009) 108, no. 3, 887–897, 10.1213/ane.0b013e31818ffd99. 19224798

[7] Abbas S. M. and Hill A. G. , Systematic Review of the Literature for the Use of Oesophageal Doppler Monitor for Fluid Replacement in Major Abdominal Surgery, Anaesthesia. (2008) 63, no. 1, 44–51, 10.1111/j.1365-2044.2007.05233.x. 18086070

[8] Bundgaard-Nielsen M. , Holte K. , Secher N. H. , and Kehlet H. , Monitoring of Peri-Operative Fluid Administration by Individualized Goal-Directed Therapy, Acta Anaesthesiologica Scandinavica. (2007) 51, no. 3, 331–340, 10.1111/j.1399-6576.2006.01221.x. 17390421

[9] Raj L. M. and Saxon L. A. , Haemodynamic Monitoring Devices in Heart Failure: Maximising Benefit with Digitally Enabled Patient Centric Care, Arrhythmia & Electrophysiology Review. (2018) 7, no. 4, 294–298, 10.15420/aer.2018.32.3. PMC6304789 30588319

[10] Brugts J. J. , Radhoe S. P. , Clephas P. R. D. et al., Remote Haemodynamic Monitoring of Pulmonary Artery Pressures in Patients With Chronic Heart Failure (MONITOR-HF): a Randomised Clinical Trial, Lancet. (2023) 401, no. 10394, 2113–2123, 10.1016/s0140-6736(23)00923-6. 37220768

[11] Kelly C. R. and Rabbani L. E. , Videos in Clinical Medicine. Pulmonary-Artery Catheterization, New England Journal of Medicine. (2013) 369, no. 25. 10.1056/NEJMvcm1212416 24350972

[12] Klabunde R. E. , Lippincott W. , and Wilkins , Cardiovascular Physiology Concepts, Philadelphia, PA Kingston upon Thames. (2022) 3rd edition, Wolters Kluwer Wolters Kluwer (UK) Ltd, Surrey.

[13] Kearney T. J. and Shabot M. M. , Pulmonary Artery Rupture Associated with the Swan-Ganz Catheter, Chest. (1995) 108, no. 5, 1349–1352, 10.1378/chest.108.5.1349. 7587440

[14] Bossert T. , Gummert J. F. , Bittner H. B. et al., Swan-Ganz catheter-induced Severe Complications in Cardiac Surgery: Right Ventricular Perforation, Knotting, and Rupture of a Pulmonary Artery, Journal of Cardiac Surgery. (2006) 21, no. 3, 292–295, 10.1111/j.1540-8191.2006.00235.x. 16684066

[15] Monnet X. and Teboul J. L. , Transpulmonary Thermodilution: Advantages and Limits, Critical Care. (2017) 21, no. 1, 10.1186/s13054-017-1739-5. PMC5474867 28625165

[16] Slagt C. , Malagon I. , and Groeneveld A. B. , Systematic Review of Uncalibrated Arterial Pressure Waveform Analysis to Determine Cardiac Output and Stroke Volume Variation, British Journal of Anaesthesia. (2014) 112, no. 4, 626–637, 10.1093/bja/aet429. 24431387

[17] Scheer B. , Perel A. , and Pfeiffer U. J. , Clinical Review: Complications and Risk Factors of Peripheral Arterial Catheters Used for Haemodynamic Monitoring in Anaesthesia and Intensive Care Medicine, Critical Care. (2002) 6, no. 3, 199–204. 10.1186/cc1489 PMC137445 12133178

[18] Sandham J. D. , Hull R. D. , Brant R. F. et al., A Randomized, Controlled Trial of the Use of Pulmonary-Artery Catheters in High-Risk Surgical Patients, New England Journal of Medicine. (2003) 348, no. 1, 5–14, 10.1056/nejmoa021108. 12510037

[19] National H. L. , Blood Institute Acute Respiratory Distress Syndrome Clinical Trials N , Wheeler A. P. et al., Pulmonary-Artery Versus Central Venous Catheter to Guide Treatment of Acute Lung Injury, New England Journal of Medicine. (2006) 354, no. 21, 2213–2224. 10.1056/NEJMoa061895 16714768

[20] Hoeper M. M. , Maier R. , Tongers J. et al., Determination of Cardiac Output by the Fick Method, Thermodilution, and Acetylene Rebreathing in Pulmonary Hypertension, American Journal of Respiratory and Critical Care Medicine. (1999) 160, no. 2, 535–541, 10.1164/ajrccm.160.2.9811062. 10430725

[21] Zhang Y. , Wang Y. , Shi J. , Hua Z. , and Xu J. , Cardiac Output Measurements via Echocardiography Versus Thermodilution: a Systematic Review and Meta-Analysis, PLoS One. (2019) 14, no. 10, 10.1371/journal.pone.0222105. PMC6776392 31581196

[22] Jozwiak M. , Monnet X. , and Teboul J. L. , Monitoring: From Cardiac Output Monitoring to Echocardiography, Current Opinion in Critical Care. (2015) 21, no. 5, 395–401, 10.1097/mcc.0000000000000236. 26348419

[23] Po J. R. , Tong M. , Meeran T. et al., Quantification of Cardiac Output with Phase Contrast Magnetic Resonance Imaging in Patients with Pulmonary Hypertension, Journal of Clinical Imaging Science. (2020) 10, 10.25259/jcis_36_2020. PMC7193209 32363088

[24] Bertaina M. , Galluzzo A. , Rossello X. et al., Prognostic Implications of Pulmonary Artery Catheter Monitoring in Patients with Cardiogenic Shock: A Systematic Review and Meta-Analysis of Observational Studies, Journal of Critical Care. (2022) 69, 10.1016/j.jcrc.2022.154024. 35344825

[25] Ju J. W. , Chung J. , Heo G. , Cho Y. J. , Jeon Y. , and Nam K. , Impact of Perioperative Pulmonary Artery Catheter Use on Clinical Outcomes After Cardiac Surgery: a Nationwide Cohort Study, Chest. (2025) 167, no. 3, 746–756, 10.1016/j.chest.2024.10.008. 39426719

[26] Watanabe A. , Miyamoto Y. , Ueyama H. , Gotanda H. , Tsugawa Y. , and Kuno T. , The Use of Pulmonary Artery Catheter and Clinical Outcomes in Older Adults With Cardiogenic Shock, International Journal of Cardiology. (2024) 417, 10.1016/j.ijcard.2024.132509. 39242035

[27] Murata Y. , Imai T. , Takeda C. , Mizota T. , and Kawamoto S. , Agreement Between Continuous Cardiac Output Measured by the fourth-generation FloTrac/Vigileo System and a Pulmonary Artery Catheter in Adult Liver Transplantation, Scientific Reports. (2022) 12, no. 1, 10.1038/s41598-022-14988-z. PMC9249899 35778523

[28] Asamoto M. , Orii R. , Otsuji M. , Bougaki M. , Imai Y. , and Yamada Y. , Reliability of Cardiac Output Measurements Using LIDCORAPID™ and FloTrac/Vigileo™ Across Broad Ranges of Cardiac Output Values, Journal of Clinical Monitoring and Computing. (2017) 31, no. 4, 709–716, 10.1007/s10877-016-9896-7. PMC5500683 27300325

[29] Mukkamala R. , Kohl B. A. , and Mahajan A. , Comparison of Accuracy of Two Uncalibrated Pulse Contour Cardiac Output Monitors in off-pump Coronary Artery Bypass Surgery Patients Using Pulmonary Artery catheter-thermodilution as a Reference, BMC Anesthesiology. (2021) 21, no. 1, 10.1186/s12871-021-01415-5. PMC8272317 34246222

[30] Senn A. , Button D. , Zollinger A. , and Hofer C. K. , Assessment of Cardiac Output Changes Using a Modified FloTrac/Vigileo Algorithm in Cardiac Surgery Patients, Critical Care. (2009) 13, no. 2, 10.1186/cc7739. PMC2689464 19261180

[31] Biancofiore G. , Critchley L. A. , Lee A. et al., Evaluation of an Uncalibrated Arterial Pulse Contour Cardiac Output Monitoring System in Cirrhotic Patients Undergoing Liver Surgery, British Journal of Anaesthesia. (2009) 102, no. 1, 47–54, 10.1093/bja/aen343. 19059920

[32] Rosenkranz S. and Preston I. R. , Right Heart Catheterisation: Best Practice and Pitfalls in Pulmonary Hypertension, European Respiratory Review. (2015) 24, no. 138, 642–652, 10.1183/16000617.0062-2015. PMC9487613 26621978

[33] Critchley L. A. and Critchley J. A. , A Meta-Analysis of Studies Using Bias and Precision Statistics to Compare Cardiac Output Measurement Techniques, Journal of Clinical Monitoring and Computing. (1999) 15, no. 2, 85–91, 10.1023/a:1009982611386. 12578081

[34] Gratz I. , Baruch M. , Awad A. , McEniry B. , Allen I. , and Seaman J. , A New Continuous Noninvasive Finger Cuff Device (Vitalstream) for Cardiac Output That Communicates Wirelessly via Bluetooth or Wi-Fi, BMC Anesthesiology. (2023) 23, no. 1, 10.1186/s12871-023-02114-z. PMC10210402 37231335

[35] Ostergaard M. , Nielsen J. , and Nygaard E. , Pulse Contour Cardiac Output: An Evaluation of the Flotrac Method, European Journal of Anaesthesiology. (2009) 26, no. 6, 484–489, 10.1097/eja.0b013e32831f343f. 19436173

[36] Della R. G. , Costa M. G. , Chiarandini P. et al., Arterial Pulse Cardiac Output Agreement with Thermodilution in Patients in Hyperdynamic Conditions, Journal of Cardiothoracic and Vascular Anesthesia. (2008) 22, no. 5, 681–687. 10.1053/j.jvca.2008.02.021 18922423

[37] Critchley L. A. , Bias and Precision Statistics: Should We Still Adhere to the 30% Benchmark for Cardiac Output Monitor Validation Studies?, Anesthesiology. (2011) 114, no. 5, 10.1097/aln.0b013e318215e1e5. 21521974

[38] Peyton P. J. and Chong S. W. , Minimally Invasive Measurement of Cardiac Output During Surgery and Critical Care: a meta-analysis of Accuracy and Precision, Anesthesiology. (2010) 113, no. 5, 1220–1235, 10.1097/aln.0b013e3181ee3130. 20881596

[39] Saugel B. , Cecconi M. , and Hajjar L. A. , Noninvasive Cardiac Output Monitoring in Cardiothoracic Surgery Patients: Available Methods and Future Directions, Journal of Cardiothoracic and Vascular Anesthesia. (2019) 33, no. 6, 1742–1752, 10.1053/j.jvca.2018.06.012. 30318422

[40] Teboul J. L. , Saugel B. , Cecconi M. et al., Less Invasive Hemodynamic Monitoring in Critically Ill Patients, Intensive Care Medicine. (2016) 42, no. 9, 1350–1359, 10.1007/s00134-016-4375-7. 27155605

[41] Penaz J. , Voigt A. , and Teichmann W. , Contribution to the Continuous Indirect Blood Pressure Measurement, Zeitschrift für die gesamte innere Medizin und ihre Grenzgebiete. (1976) 31, no. 24, 1030–1033. 1020409

[42] Bogert L. W. , Wesseling K. H. , Schraa O. et al., Pulse Contour Cardiac Output Derived from Non-invasive Arterial Pressure in Cardiovascular Disease, Anaesthesia. (2010) 65, no. 11, 1119–1125, 10.1111/j.1365-2044.2010.06511.x. 20860647

[43] Fischer M. O. , Avram R. , Carjaliu I. et al., Non-Invasive Continuous Arterial Pressure and Cardiac Index Monitoring with Nexfin After Cardiac Surgery, British Journal of Anaesthesia. (2012) 109, no. 4, 514–521, 10.1093/bja/aes215. 22750726

[44] Nelson M. R. , Stepanek J. , Cevette M. , Covalciuc M. , Hurst R. T. , and Tajik A. J. , Noninvasive Measurement of Central Vascular Pressures with Arterial Tonometry: Clinical Revival of the Pulse Pressure Waveform?, Mayo Clinic Proceedings. (2010) 85, no. 5, 460–472, 10.4065/mcp.2009.0336. PMC2861976 20435839

[45] Zayat R. , Goetzenich A. , Lee J. Y. et al., Comparison Between Radial Artery Tonometry Pulse Analyzer and pulsed-Doppler Echocardiography Derived Hemodynamic Parameters in Cardiac Surgery Patients: a Pilot Study, PeerJ. (2017) 5, 10.7717/peerj.4132. PMC5723137 29230366

[46] Wagner J. Y. , Sarwari H. , Schon G. et al., Radial Artery Applanation Tonometry for Continuous Noninvasive Cardiac Output Measurement: A Comparison With Intermittent Pulmonary Artery Thermodilution in Patients After Cardiothoracic Surgery, Critical Care Medicine. (2015) 43, no. 7, 1423–1428, 10.1097/ccm.0000000000000979. 25844700

[47] Fellahi J. L. and Fischer M. O. , Electrical Bioimpedance Cardiography: an Old Technology with New Hopes for the Future, Journal of Cardiothoracic and Vascular Anesthesia. (2014) 28, no. 3, 755–760, 10.1053/j.jvca.2013.12.026. 24917062

[48] Marik P. E. , Noninvasive Cardiac Output Monitors: A State-of the-Art Review, Journal of Cardiothoracic and Vascular Anesthesia. (2013) 27, no. 1, 121–134, 10.1053/j.jvca.2012.03.022. 22609340

[49] Joosten A. , Desebbe O. , Suehiro K. et al., Accuracy and Precision of Non-Invasive Cardiac Output Monitoring Devices in Perioperative Medicine: A Systematic Review and Meta-Analysisdagger, British Journal of Anaesthesia. (2017) 118, no. 3, 298–310, 10.1093/bja/aew461. 28203792

[50] Recco D. P. , Roy N. , Gregory A. J. , and Lobdell K. W. , Invasive and Noninvasive Cardiovascular Monitoring Options for Cardiac Surgery, JTCVS Open. (2022) 10, 256–263, 10.1016/j.xjon.2022.02.028. PMC9390282 36004243

[51] Broch O. , Renner J. , Gruenewald M. et al., A Comparison of the Nexfin(R) and Transcardiopulmonary Thermodilution to Estimate Cardiac Output During Coronary Artery Surgery, Anaesthesia. (2012) 67, no. 4, 377–383, 10.1111/j.1365-2044.2011.07018.x. 22324797

[52] Wagner J. Y. , Korner A. , Schulte-Uentrop L. et al., A Comparison of Volume Clamp Method-Based Continuous Noninvasive Cardiac Output (CNCO) Measurement Versus Intermittent Pulmonary Artery Thermodilution in Postoperative Cardiothoracic Surgery Patients, Journal of Clinical Monitoring and Computing. (2018) 32, no. 2, 235–244, 10.1007/s10877-017-0027-x. 28540614

[53] Maass S. W. , Roekaerts P. M. , and Lance M. D. , Cardiac Output Measurement by Bioimpedance and Noninvasive Pulse Contour Analysis Compared with the Continuous Pulmonary Artery Thermodilution Technique, Journal of Cardiothoracic and Vascular Anesthesia. (2014) 28, no. 3, 534–539, 10.1053/j.jvca.2014.01.007. 24746334

[54] Castaneda D. , Esparza A. , Ghamari M. , Soltanpur C. , and Nazeran H. , A Review on Wearable Photoplethysmography Sensors and Their Potential Future Applications in Health Care, International Journal of Biosensors & Bioelectronics. (2018) 4, no. 4, 195–202. 10.15406/ijbsbe.2018.04.00125 PMC6426305 30906922

[55] Panula T. , Sirkia J. P. , Wong D. , and Kaisti M. , Advances in Non-Invasive Blood Pressure Measurement Techniques, IEEE Reviews in Biomedical Engineering. (2023) 16, 424–438, 10.1109/rbme.2022.3141877. 35015647

[56] Nachman D. , Eisenkraft A. , Goldstein N. et al., Influence of Sex, BMI, and Skin Color on the Accuracy of Non-Invasive Cuffless Photoplethysmography-Based Blood Pressure Measurements, Frontiers in Physiology. (2022) 13, 10.3389/fphys.2022.911544. PMC9277111 35846008

[57] Nachman D. , Gepner Y. , Goldstein N. et al., Comparing Blood Pressure Measurements Between a Photoplethysmography-Based and a Standard Cuff-based Manometry Device, Scientific Reports. (2020) 10, no. 1, 10.1038/s41598-020-73172-3. PMC7527983 32999400

[58] Nachman D. , Constantini K. , Poris G. et al., Wireless, Non-Invasive, Wearable Device for Continuous Remote Monitoring of Hemodynamic Parameters in a Swine Model of Controlled Hemorrhagic Shock, Scientific Reports. (2020) 10, no. 1, 10.1038/s41598-020-74686-6. PMC7573605 33077774

[59] Kachel E. , Constantini K. , Nachman D. et al., A Pilot Study of Blood Pressure Monitoring After Cardiac Surgery Using a Wearable, Non-invasive Sensor, Frontiers of Medicine. (2021) 8, 10.3389/fmed.2021.693926. PMC8375406 34422859

[60] Nachman D. , Gilan A. , Goldstein N. et al., Twenty-Four-Hour Ambulatory Blood Pressure Measurement Using a Novel Noninvasive, Cuffless, Wireless Device, American Journal of Hypertension. (2021) 34, no. 11, 1171–1180. 10.1093/ajh/hpab095 34143867

[61] Dvir A. , Goldstein N. , Rapoport A. et al., Comparing Cardiac Output Measurements Using a Wearable, Wireless, Noninvasive Photoplethysmography-Based Device to Pulse Contour Cardiac Output in the General ICU: A Brief Report, Critical Care Explorations. (2022) 4, no. 2, 10.1097/cce.0000000000000624. PMC8812679 35128457

[62] Nachman D. , Eisenkraft A. , Rahamim E. et al., Assessing Cardiac Flow Measurements Using a Noninvasive Photoplethysmography-Based Device Compared to Invasive Pulmonary Artery Catheter, JACC Advances. (2025) 4, no. 9, 10.1016/j.jacadv.2025.102093. PMC12398815 40845743

[63] Fares W. H. , Blanchard S. K. , Stouffer G. A. et al., Thermodilution and Fick Cardiac Outputs Differ: Impact on Pulmonary Hypertension Evaluation, Cancer Research Journal. (2012) 19, no. 4, 261–266, 10.1155/2012/261793. PMC3411391 22891186

[64] Opotowsky A. R. , Hess E. , Maron B. A. et al., Thermodilution vs Estimated Fick Cardiac Output Measurement in Clinical Practice: an Analysis of Mortality from the Veterans Affairs Clinical Assessment, Reporting, and Tracking (VA CART) Program and Vanderbilt University, JAMA Cardiology. (2017) 2, no. 10, 1090–1099, 10.1001/jamacardio.2017.2945. PMC5710449 28877293

[65] Tehrani D. M. , Grinstein J. , Kalantari S. et al., Cardiac Output Assessment in Patients Supported with Left Ventricular Assist Device: Discordance Between Thermodilution and Indirect Fick Cardiac Output Measurements, ASAIO Journal. (2017) 63, no. 4, 433–437, 10.1097/mat.0000000000000528. PMC5489370 28125464

